# Medical genomics research at BGRS-2018

**DOI:** 10.1186/s12920-019-0480-0

**Published:** 2019-03-13

**Authors:** Ancha V. Baranova, Vadim V. Klimontov, Andrey Y. Letyagin, Yuriy L. Orlov

**Affiliations:** 1 School of Systems Biology, George Mason University, Fairfax, VA USA; 2 Research Centre for Medical Genetics, Moscow, 115478 Russia; 3 Institute of Cytology and Genetics SB RAS, 630090 Novosibirsk, Russia; 4 Novosibirsk State University, 630090 Novosibirsk, Russia

This Special Issue of BMC Medical Genomics collates the papers presented at the biannual summit in Bioinformatics and Systems Biology BGRS\SB (Bioinformatics of Genome Regulation and Structure\Systems Biology) – 2018 (http://conf.bionet.nsc.ru/bgrssb2018/en/). This international conference takes place in Novosibirsk since 1998. To accompany this Special Issue, other Special Issues in the fields of genomics, bioinformatics, plant biology, evolutionary biology and systems biology are published as a part of following series: BMC Genomics, BMC Bioinformatics, BMC Systems Biology, BMC Genetics, BMC Evolutionary Biology and BMC Plant Biology [1–4]. In year 2017, respective highlights were organized into the Special Issues with reports from Belyaev Readings-2017 (http://conf.bionet.nsc.ru/belyaev100/en) [5–8].

The papers comprising this issue of BMC Medical Genomics were discussed at the BGRS\SB–affiliated symposium “Systems Biology and Biomedicine” (SBioMed-2018) (http://conf.bionet.nsc.ru/ishg2018/en/). A brief summary of these papers is outlined below.

We open up this Special Issue with an interesting report of a negative finding. After applying a variety of high-throughput technologies such as whole-exome sequencing, transcriptome and miRNA analysis, Alexander Lavrov [9] and his team did not find any reliable molecular markers for early prediction of primary resistance or intolerance to imatinib in adult patients with chronic myeloid leukemia (CML). This report contributes to the body of the literature demonstration a lack of consensus between the signatures describing primary responders and non-responders among CML patients [10, 11] and points at shortcoming of current approaches [12] for identifying diagnostic and prognostic biomarkers of human diseases.

The following papers utilized high-throughput biomarker discovery approach for extracting insights concerning tumorigenesis. Jun Lu and co-authors [13] demonstrated that the treatment of non-small cell lung cancer (NSCLC) with anlotinib, a promising tyrosine kinase inhibitor that targets vascular endothelial growth factor receptor (VEGFR), fibroblast growth factor receptor (FGFR), platelet-derived growth factor receptors (PDGFR), and c-kit, may result in the development of resistance due to induction of macrophage inflammatory protein 2-alpha cytokine encoding gene *CXCL2*, which is involved in wound healing, cancer metastasis, and angiogenesis. Anna Kudryavtseva et al. [14] continue discussion on cancer studies by analysis of mutations in carotid body tumor. Carotid body tumor (CBT) is a rare neoplasm arising from carotid paraganglion in the head and neck [15]. This tumor is heterogeneous and could be associated with both germline and somatic variants. Molecular mechanisms of CBT development are not fully understood. The authors performed exome sequencing of tumors with paired lymph node tissues and peripheral blood obtained from the CBT patients. Mutation load was estimated as the number of somatic variants per megabase of the target regions covered by the Illumina library preparation kit. This work continues previous studies of the authors group on cancer cells presented after Belyaev conference - 2016 [16]. Here Kudryavtseva and colleagues estimated mutation spectra and identified pathogenic somatic and germline variants in the patients.

Elena Voropaeva et al. [17] comprehensively explored variability in *TP53* in samples of diffuse large B-cell lymphoma (DLBCL) to show that two sequential inactivation events within that well-known tumor-suppressor locus are required for malignant transformation.

Darya Skuratovskaya and her colleagues [18] profiled mtDNA copy number in various adipose tissue compartments and in peripheral blood mononuclear cells (PBMCs) of obese individuals with and without type 2 diabetes to show a possibility that the influence of proinflammatory factors may engage compensatory increases in mitochondrial metabolism. On the other hand, elevation of mtDNA copy number was associated with a decrease in secretion of chemerin, known adipokine implicated in autocrine / paracrine signaling stimulating lipolysis. The issue is complicated by the relationship between mtDNA copy number in insulin-dependent tissues to the levels of the biomarkers of endothelial dysfunction and inflammation, which was uncovered by Larisa Litvinova and co-authors [19]. Their work shows that in patients with obesity, systemic subclinical inflammation is paralleled with an increase in mtDNA copies and underlying disturbances of carbohydrate and lipid metabolism.

Peter Sparber and co-authors [20] performed a comprehensive review of involvement of long non-coding RNAs in hereditary diseases in humans, with an emphasis on their mechanistic connections to the development of particular pathophysiological processes spanning OMIM entities from well-known Mendelian disorders to the diseases of imprinting and to the common age-associated conditions like Alzheimer’s. Authors thoroughly review a role of pathogenesis-associated lncRNA in the recruitment of chromatin-modifying complexes, antisense transcription, splicing regulation, miRNA processing, RNA-RNA duplex formation and others.

Nikolay Zernov and Mikhail Skoblov [21] presented thorough review of genotype-phenotype correlations in facio-scapulo-humeral muscular dystrophy (FSHD), which is due ectopic expression of the transcription factor DUX4 in skeletal muscles, and identified a set of genetic and epigenetic modifiers for this condition. Understanding of the systems biology of this important condition is crucial for the success of future clinical trials.

Sergey Kulemzin and colleagues [22] set upon increasing our understanding of the mechanisms of chimeric antigen receptors (CAR) activation in T and NK cells by comparing cell-specific functionalities of several activation-inducible promoters. In activated T cells, a variant of CD69 promoter was pinpointed as the most potent driver of the expression of engineered mRNAs, while 10xNFkB performed in T and NK cell with equal power. Unfortunately, the latter promoter has also leaked in absence of stimulation, thus, remaining a suboptimal choice for expression of exogenous sequences. Hence, the search for NK-cell suitable activation-inducible promoter configurations is not yet over.

Daria Kashirina and other members of international team [23] assembled by Irina Larina and Eugene Nikolaev performed quantitative analysis of blood proteins in samples collected at various stages of space flight. Notably, the levels of calprotectin S100A9, a protein with an important role in the functioning of the endothelium, have increased after the landing, in parallel with that for the proteins of complement coagulation cascade, the acute phase reactants, and the proteases. It seems that acute re-adaptation to the gravity activates an inflammatory response in cosmonauts’ hypotrophic muscles.

Vladimir Babenko and colleagues [24] analyzed the *FTO* gene locus for its haplotype profiles within eighteen 1000G populations from 4 continental groups. Their study points that this obesity-associated locus evolved rapidly, confirming previously reported evidence of the selective sweep [25], which took place in the transcription factors binding sites-enriched sequences within intron 3 of *FTO*.

Olga Saik and co-authors [26] employed an Associative Network Discovery System for automated literature mining in the field of biology (ANDSystem), previously described by the same group of authors [27] to prioritize the genes involved in endothelial apoptosis and contributing to lymphedema, an debilitating swelling which is due to defects in lymphatic drainage. There is a hope that experimental analysis of the function of these genes will aid in understanding the lymphedema and development of targeted treatments.

Darya Telegina et al. [28] used well-known strain of senescence-accelerated OXYS rats model [29] to study age-related macular degeneration (AMD) through comparative profiling NGF, BDNF, and their receptors for immunohistochemical localization of in a normal and AMD-like rat retinas, and to confirm that alterations in the balance of neurotrophic factors contribute to the development of AMD.

Hence, we present our readers with a wide array of reports describing recent breakthrough in genomics-driven understanding of a molecular pathophysiology of a variety of human disease, covering a spectrum from Mendelian disorders to chronic multifactorial conditions to cancer. At BGRS-2018, the symposium “Systems Biology and Biomedicine” (SBioMed-2018) was also attended by young scientists who gathered in Novosibirsk for a School “Systems Biology and Bioinformatics” (SBB-2018) (http://conf.bionet.nsc.ru/bgrssb2018/en/school/). In previous years, the materials of SBB Schools were published in Special Issues of BMC as well [30]. We invite our readers worldwide to attend our next event - Systems Biology and Bioinformatics Young Scientists School (SBB-2019) which will be held in Novosibirsk, Russia some time during summer of 2019 (http://conf.bionet.nsc.ru/sbb2019/en/). Medical genomics and genetics researches will be discussed as well at VII Congress of Vavilov Society of Geneticists and Breeders at St.-Petersburg State University in June 2019 (https://events.spbu.ru/events/genetic-selection-2019). Next year conference BGRS\SB-2020 in Novosibirsk is scheduled to June 2020.
